# Ligature of the Posterior Tibial Artery for a Wound

**Published:** 1846-05

**Authors:** John Charles Hall

**Affiliations:** Member of the Royal College of Surgeons, of England, &c. &c.


*Ligature of the Posterior Tibial Artery for a Wound.*—By John Charles Hall, M. D., Member of the Royal College of Surgeons, of England, &c. &c.—I have read with great interest and attention the valuable paper forwarded to the Royal Medical and Chirurgical Society, by Mr. Arnott, and also the remarks made by several of the experienced surgeons who entered into an examination of the subject in the discussion to which the paper gave rise. It appears that a wound of this artery is not a very frequent occurrence, evidenced by the fact that Mr. B. Cooper, in his very extensive experience, had only seen two cases; I may, therefore, perhaps be permitted to forward my notes of a case in which this artery was tied by myself some years ago.

April 16th, 1841.—I was requested by Mr. Parkinson to visit, with all possible haste, Mr. N——, a gentleman upwards of sixty, who had been thrown from his horse, and was, to use the words of Mr. P., "bleeding to death from a wound of the leg." As I was within a mile of Mr. N.'s house at the time of the accident, the case was seen in less than half an hour after the infliction of the injury. His clothes were saturated with blood, and a considerable quantity had flowed into his shoe. He was cold and faint, with a feeble purse. A little brandy and water was immediately given to him, and he was placed in bed. On removing his clothes, a wound of about an inch in length was seen in the calf, a little below the junction of the upper with the middle third, and from which the blood was now again beginning to flow freely. A tourniquet was applied to the femoral artery, which retarded the hæmorrhage, but did not altogether check it. The nature of the injury was at once evident, and the necessary steps immediately taken for securing the posterior tibial artery. Introducing a long narrow-bladed knife into the wound, the muscles were carefully divided above and below the seat of injury, down to the deep fascia which was now clearly seen on sponging away the coagulated blood, and into which a small opening had been made. (The manner in which the injury had been sustained was doubtful. At the time, the old gentleman was riding a horse that plunged violently and threw him with great force to the ground ; his foot was fixed in the stirrup, and he was dragged some distance ; he says when his foot got at liberty the horse kicked him with considerable violence, on the leg; he had neither trowsers nor gaiters upon his leg, which was only protected by a thick stocking. On looking at the shoes of the horse, we found he had been shod behind with what are called "*caulkings*," one of which Mr. N. supposed had been driven into his leg.)

Enlarging the opening in the fascia, after removing a good deal of coagulated blood, the artery was found, and secured by passing a ligature above and below the wound and dividing the vessel between them. The wound in the muscles was then brought together, and a bandage passed from the toes to the upper part of the thigh ; the patient appeared to experience much relief from this, which, in a great measure, checked the spasm and quivering of the muscles, which were somewhat severe during and immediately after the operation. A large dose of Liq. Opii. Sed. was given. No constitutional disturbance worthy of notice took place. Both ligatures came away on the 12th day after the operation. I saw Mr. N. a few days ago ; he was on horse-back, and told me he could not discover that the leg on which the injury was sustained was worse than the other ; and that it did its work very well.

Mr. B. Cooper appears to think that the other operation is better, and that the artery can be readily found and secured by separating the *soleus* from its attachment to the tibia. I know not how this can be managed on the living subject, but I well know its difficulties on the dead ; and remember after trying it several times on the dead body remarking to my esteemed friend and old teacher, Mr. Lane, that I never dare venture to take up the artery in this manner, if called to a patient with a wound of the posterior tibial artery. Mr. Guthrie, in his usual graphic manner, has thus stated the difficulty of thus performing the operation. " The operator has cut his four inches, has turned up the head of the *gastrocnemius*, and has insinuated his director under the head of the *soleus*, which he has also *sliced away from the bone*, the artery is still an inch inwards, bound down by a strong fascia, which must be cut immediately over or by the side of the artery ; it will not do to separate it from the bone and then push it over ; it cannot be done." Even when this fascia is divided after the most approved fashion, Guthrie considers it almost impracticable to separate the two veins from the artery, and to pass a ligature under it from without inwards, so as to avoid the nerve. " If a bystander should inquire *why this most difficult, painful, bloody, tedious, and dangeroug operation is done ?* the answer would be, solely because it was not usual to make a longitudinal incision in the muscles of the calf of the leg —an incision which, if made by accident, would be pronounced to be one attended with little danger, and not likely to lead to any subsequent detriment. " (*Guthrie on Diseases and Injuries of the Arteries*, p. 259.)

With regard to the point alluded to by Mr. Cæsar Hawkins, "that it was desirable to secure the artery above the wound in cases in which we were ignorant of its precise direction," he mentions a case in which he could not tell whether the femoral or profunda was wounded ; he therefore placed a ligature on the common femoral, and the patient recovered. In a case that came under my care, the particulars of which are stated in this journal (April 26, 1844,) in which the patient had received a stab with a long knife at the upper part of the thigh, it was necessary to apply a ligature to the common femoral above the part where it was wounded, another below, and another below that part of the profunda which was wounded, before the bleeding was arrested. It appears, I think, the proper rule of practice always, if called to a patient *immediately after* the injury has been sustained, to enlarge the wound, and to tie the artery above and below the part that has been opened.

Mr. Hodson mentions a case in which death took place, where the brachial artery being wounded, a ligature was only placed above the part injured. The loss of blood which deprived the unfortunate patient of life was from the lower end; and this person's life would, in all probability, not have been sacrificed, had both ends of the vessel been secured on the receipt of the wound. (On Disease of Arteries, page 469.) Mr. Liston says, "it is not sufficient to include the vessel above the wounded part, for the lower part will, after some time, be supplied by the collateral branches almost as freely as by the large trunk, and consequently bleeding will be renewed; two ligatures are to be employed, one above, and the other below the wound," (page 236.) Mr. Guthrie is of opinion, that the lower end of a divided artery is more prone to secondary hæmorrhage than the upper, and that the retraction and contraction of the lower end of a divided artery is neither so perfect nor so permanent as at the upper end, and that the internal coagulum is in many instances altogether wanting or very defective in its formation (p. 251.)

In the well-known case of Mr. S. Cooper, in which he took up the femoral artery in the middle of the thigh, when the popliteal had given way ten days after the passage of a musket-ball through the ham, no doubt the inflammation in the ham had plugged up the lower end of the artery; and in the case in which this gentleman secured the popliteal artery for a wound of the posterior tibial, there is no doubt but the "extensive ill-conditioned wound" made by the pane of glass, and the "ineffectual attempts of the house-surgeon to secure the posterior tibial," led to the same result, with regard to the lower portion of the wounded vessel. Mr. Liston concludes his argument in favour of cutting down upon a wounded artery, and securing it above and below the opening made in it, thus—"*Cases of hæmorrhage have occurred in which the tying the vessel immediately above the wound has been successful, but these are few, and by no means afford any argument for the adoption of such a measure.*"

Lastly, I venture to express the opinion, that the fears of surgeons that the divided muscular fibres will not unite, are in a great measure groundless; at any rate this case adds nothing to those already recorded, in which the posterior tibial artery has been secured in the way recommended by Mr. Guthrie,—in which the wound in the muscles has healed favourably, and the patient completely recovered.—*London Medical Gazette.*

